# Supplementary material for: Polyketide synthase gene clusters in the ericoid mycorrhizal fungus Oidiodendron maius: genome-wide prediction, expression profiling, and functional characterization of knockout mutants
Source: Front Plant Sci. 2026 Sep 16;17:1924430. doi: 10.3389/fpls.2026.1924430 (PMC13623903; doi:10.3389/fpls.2026.1924430)
Supplement: Supplementary file 1 [file SupplementaryFile1.zip › Supplementary Materials/Supplementary Folder 1.zip!/Oidma1_AssemblyScaffolds_Repeatmasked/knownclusterblast/cluster35/jgi.p_Oidma1_32800_mibig_hits.html]

| MIBiG Protein | Description | MIBiG Cluster | MiBiG Product | % ID | % Coverage | BLAST Score | E-value |
| --- | --- | --- | --- | --- | --- | --- | --- |
| EGD99349.1 | dimethylallyl\_tryptophan\_synthase | BGC0001144 | Neosartoricin\_biosynthetic\_gene\_cluster | 54 | 98.6486486486 | 495 | 8.4e-140 |
| ADI24928.1 | VrtC | BGC0000168 | Viridicatumtoxin\_biosynthetic\_gene\_cluster | 52 | 98.6486486486 | 448 | 6.9e-126 |
| CBF80712.1 | Putative\_uncharacterized\_proteinTdiB\_;\_[Source:UniProtKB/TrEMBL;Acc:Q5AT66] | BGC0000442 | Terrequinone\_biosynthetic\_gene\_cluster | 23 | 81.7567567568 | 61 | 3.1e-09 |
